# Eosinophilic esophagitis prevalence in adults with dysphagia or heartburn: preliminary results

**DOI:** 10.1186/1939-4551-8-S1-A251

**Published:** 2015-04-08

**Authors:** Jose Laerte Boechat, Beatriz Biccas, Boris Medrano, Daniella Moore

**Affiliations:** 1 Universidade Federal Fluminense, Brazil

## Background/aim

Eosinophilic esophagitis (EoE) is a chronic immune-mediated condition characterized clinically by esophageal dysfunction and pathologically by infiltration of > 15 eosinophils per high-powered field (HPF) in the esophageal mucosa, since other causes of esophageal eosinophilia (including proton-pump inhibitor responsive esophageal eosinophilia [PPI-REE]) are excluded. It occurs worldwide especially in children and adults < 40 years, affecting notably Caucasian male. Treatment includes dietary elimination of food allergens and topical corticosteroids.

The aim of this study is to determine the prevalence of EoE in adults with dysphagia or heartburn followed in the Immunology and Gastroenterology Services of the Hospital Universitario Antonio Pedro, Universidade Federal Fluminense, Niteroi, Brazil.

## Methods

Prospective study. Patients complaining of heartburn for more than twice a week, dysphagia or food impaction were submitted to a clinical questionnaire and upper gastrointestinal endoscopy (UGE) to exclude specific causes. If the chief complaint was dysphagia and UGE was normal or revealed typical EoE features, biopsy was performed in proximal, medial and distal esophagus, gastric antrum and duodenum. For the ones with heartburn and erosive esophagitis or normal UGE, treatment with PPI was offered for 8 weeks and biopsy was also performed in case of non response. Patients with dysphagia and histological criteria of EoE (> 15 eosinophils/HPF in the esophagus only) received PPI for 8 weeks and repeated biopsy to distinguish EoE from PPI-REE. Skin prick tests (SPT) with aeroallergens and foods were performed in patients with EoE diagnosis.

## Results

A total of 77 subjects were enrolled (79,2% female, mean age 59,4 yo [22-83 yo], 49,4% with dysphagia, 50,6% heartburn). Seventeen (22,1%) were examined by biopsy which revealed at least 15 eosinophils per HPF in 3 cases (3.8%), all of them with typical endoscopic findings of EoE. One of those was confirmed with EoE (prevalence of 1,3%), the other was a PPI-REE and the last patient waits histopathological confirmation. SPT were negative in the patient with EoE.

## Conclusion

The prevalence of EoE among brazilian adults with dysphagia or heartburn was 1,3% (1 of 77 patients), similar to the low prevalence reported in international literature.

